# Development of colorimetric and machine learning based accurate glucose detection platform for point of care applications

**DOI:** 10.1038/s41598-026-54054-6

**Published:** 2026-05-20

**Authors:** Mithun Kanchan, Pragna Harish, Tushit Chatterjee, Omkar S Powar

**Affiliations:** 1 Manipal Institute of Technology, Manipal Academy of Higher Education, Manipal, Karnataka 576104 India; 2 Manipal Institute of Technology, Manipal Academy of Higher Education, Manipal, Karnataka 576104 India

**Keywords:** Machine learning, Point-of-Care, Glucose, Microfluidics, Image processing, Biomedical engineering, Mechanical engineering, Health care

## Abstract

Improving overall health and preventing complications is crucial for timely and effective treatment of diabetes patients. In this direction, accurate measurement and detection of glucose concentration in blood is essential. The aim of this study is to develop an affordable, reliable and accurate Point-of-Care (POC) diagnostic platform for glucose concentration detection using microfluidic and colorimetric principles. A microfluidic chip is fabricated which provides the base for efficient colorimetric reaction. The proposed system requires only ~ 20 µL of sample per microwell, with a colorimetric reaction time of 3–4 min. The chip is positioned inside a compact, USB-powered, 3D printed image capture device affixed with a high-resolution camera so that variables such as camera position, focal distance and lighting conditions can be controlled. The analysis workflow is adaptable for integration with embedded systems or laptops, making it suitable for real-time deployment in Point-of-Care settings without the need for smartphones or calibration tools. The images captured inside the device (1280 in total, corresponding to 16 glucose concentration levels ranging from 50 to 200 mg/dL) were labeled based on known concentration levels and subjected to standardized preprocessing. Key preprocessing steps included Region of Interest (ROI) extraction using a fixed box detection algorithm, normalization of pixel values, image resizing to 128 × 128 pixels, and standardization using ImageNet parameters. These ensured uniformity and robustness prior to feature extraction and machine learning classification. The classifiers used are Decision Tree (DT), Random Forest (RF), Support Vector Machine (SVM), Neural Networks (NN), and K-Nearest Neighbours (KNN). The RF machine learning classifier achieved the highest cross-validation accuracy of 87.47% and precision of 89.14%, demonstrating its ability to effectively distinguish between different glucose concentration levels. The confusion matrix and ROC curve analysis further validated the model’s robustness, with minimal misclassifications and a high mean AUC value of 0.9509. The results thus highlight the potential of image-based glucose concentration estimation as a cost-effective and scalable solution for real-time monitoring in biomedical applications.

**Supplementary Information:**

The online version contains supplementary material available at 10.1038/s41598-026-54054-6.

## Introduction

Diabetes mellitus (DM) is a metabolic disorder characterized by abnormal fluctuations in blood glucose levels. It primarily results from insufficient insulin production or impaired insulin function, which can lead to serious systemic complications. According to the World Health Organization (WHO), more than 95% of people with diabetes have type 2 diabetes, which can cause extensive damage to the nervous and vascular systems. Chronic hyperglycemia is a leading cause of blindness, renal failure, myocardial infarction, stroke, and lower-limb amputation. Therefore, timely and continuous glucose monitoring is essential for effective diabetes management.

Colorimetric detection has long been recognized as a reliable and cost-effective approach for glucose estimation, where the color intensity generated by the enzymatic reaction is directly correlated with glucose concentration^1–3^. The color intensity is traditionally quantified spectrophotometrically, yielding highly accurate concentration measurements. Recent advances in Point-of-Care (POC) diagnostics have introduced microfluidic platforms that enable rapid, efficient, and low-cost colorimetric analysis, particularly valuable in resource-limited environments^4–6^. Such devices improve sensitivity, reduce sample and reagent volumes, and simplify user operation, making them ideal for field-deployable diagnostics. When developing a colorimetric-based POC glucose detection device, three key aspects must be carefully considered. First, the device should provide a stable platform capable of reliably supporting the enzymatic colorimetric reaction. Second, it must incorporate an optical or imaging mechanism that can precisely capture and quantify the color intensity of the reaction product. Third, an appropriate calibration or computational technique is required to ensure analytical accuracy, linearity, and sensitivity across varying glucose concentration ranges.

Paper-based microfluidic analytical devices (µPADs) have gained significant popularity for colorimetric assays in recent years^7–16^. These devices can be fabricated using diverse methods such as wax printing, inkjet printing, photolithography, laser or plasma treatment, and screen printing. Although µPADs offer advantages including low cost, ease of use, portability, and disposability, they also exhibit limitations such as sample overflow, washing effects, non-uniform color formation due to uneven enzyme immobilization, multi-step fabrication, surface-treatment dependency, and porosity-related inconsistencies. These challenges can be effectively mitigated by adopting Xurography, a rapid-prototyping technique that employs a razor cutter or cutting plotter to create microstructures in thin polymer films^17^. Xurography allows fast, inexpensive, and reproducible fabrication of microfluidic devices^18,19^, enabling applications in cell culture^20^, biosensing, and educational laboratories^21^. Recent POC glucose detection strategies increasingly combine smartphone-based imaging with machine-learning (ML) algorithms to improve analytical accuracy and device portability^22–31^. Mercan et al.^32^ demonstrated glucose detection in artificial saliva using a smartphone-coupled µPAD, where colorimetric analysis and ML interpretation enabled rapid and user-friendly glucose estimation. Similarly, Lee et al.^33^ developed a regression-based ML approach for simultaneous pH and glucose detection using colorimetric paper sensors. Extracted color features from RGB and HSV spaces were fed into ML models to predict glucose concentrations, demonstrating promising accuracy and cost-effectiveness. Kılıc et al.^34^ introduced a non-enzymatic glucose detection method using Au/Ag nanoparticles and smartphone imaging, eliminating the stability issues associated with enzymatic systems. The researchers optimized lighting conditions and standardized the camera-sample distance to minimize variability. Hue and intensity shifts were analyzed, and both linear and non-linear ML models were trained to predict glucose concentrations based on colorimetric responses. Ghateii and Jahanshahi^35^ proposed a flash/no-flash imaging technique combined with ML models to mitigate environmental illumination inconsistencies in glucose monitoring. Their Android application - *Gluco Estimator* - processed colorimetric changes using two distinct indicators (TMB for low and KI for high glucose levels), achieving high classification accuracy (0.95 and 0.91, respectively) across a glucose concentration range of 0–30 mM. Poddar et al.^36^ presented a glucose monitoring system employing enzymatic glucose oxidase immobilized on a substrate, with smartphone-captured images processed for noise reduction, color correction, and feature extraction. The extracted color data were converted into numerical values, and pattern recognition algorithms were utilized to detect glucose-induced color changes, ensuring reliable quantification. Recently, Kanchan et al.^37,38^ developed a Convolution Neural Network (CNN) model to predict concentration of glucose on a microfluidic analytical device and Choi et al.^39^ performed transfer learning and data augmentation for glucose concentration prediction. Despite these advancements, real-world deployment of smartphone-assisted ML glucose sensors remains challenging, primarily due to variability in smartphone cameras, optical geometries, ambient lighting, and calibration-curve dependence. These factors, together with the need for advanced image-correction algorithms, often limit reproducibility and quantitative reliability^40^.

To address these challenges, the present study focuses on the development of a cost-effective and efficient microfluidic analytical chip constructed using a glass baseplate layered with adhesive-coated polyvinyl films. These films are precisely patterned using a cutting plotter and stacked together, resulting in a novel microfluidic device optimized for enzyme–reagent interactions, specifically tailored for colorimetric glucose detection. This fabrication approach enables rapid prototyping and enhances user convenience. The microfluidic chip is housed within a custom-designed 3D-printed, camera-integrated imaging module, ensuring controlled lighting conditions and a fixed focal distance. Previous studies have investigated the use of ML models for image processing and analysis; however, most rely on manual feature extraction, where key image parameters are identified and compared across different ML algorithms to determine the most effective model for a given application. These models have demonstrated strong capabilities in pattern recognition, image classification, and object detection. The ML models employed in this study are computationally lightweight shallow-learning algorithms, including Decision Tree, Random Forest, Support Vector Machine (SVM), K-Nearest Neighbors (KNN), and a basic Feedforward Neural Network (FNN). These models were trained on statistical features extracted from images—such as mean, standard deviation, skewness, and entropy—resulting in a low-dimensional and informative feature matrix. Due to this simplified feature representation and model architecture, each model required less than five minutes for training, and inference time per sample remained well under 100 milliseconds, highlighting the computational efficiency and suitability of the system for real-time deployment in POC diagnostic settings. This approach eliminates the need for external calibration curves typically established under controlled laboratory conditions by directly mapping extracted image features to verified glucose concentration classes, enabling reliable analyte estimation. Our study introduces a fixed-camera-based colorimetric detection system that functions as a standalone instrument, eliminating dependence on external components such as smartphones, casings, or additional holding platforms, thereby providing a robust, portable, and user-friendly POC solution.

## Methodology

### Microfluidic chip fabrication

**Fig. 1:**
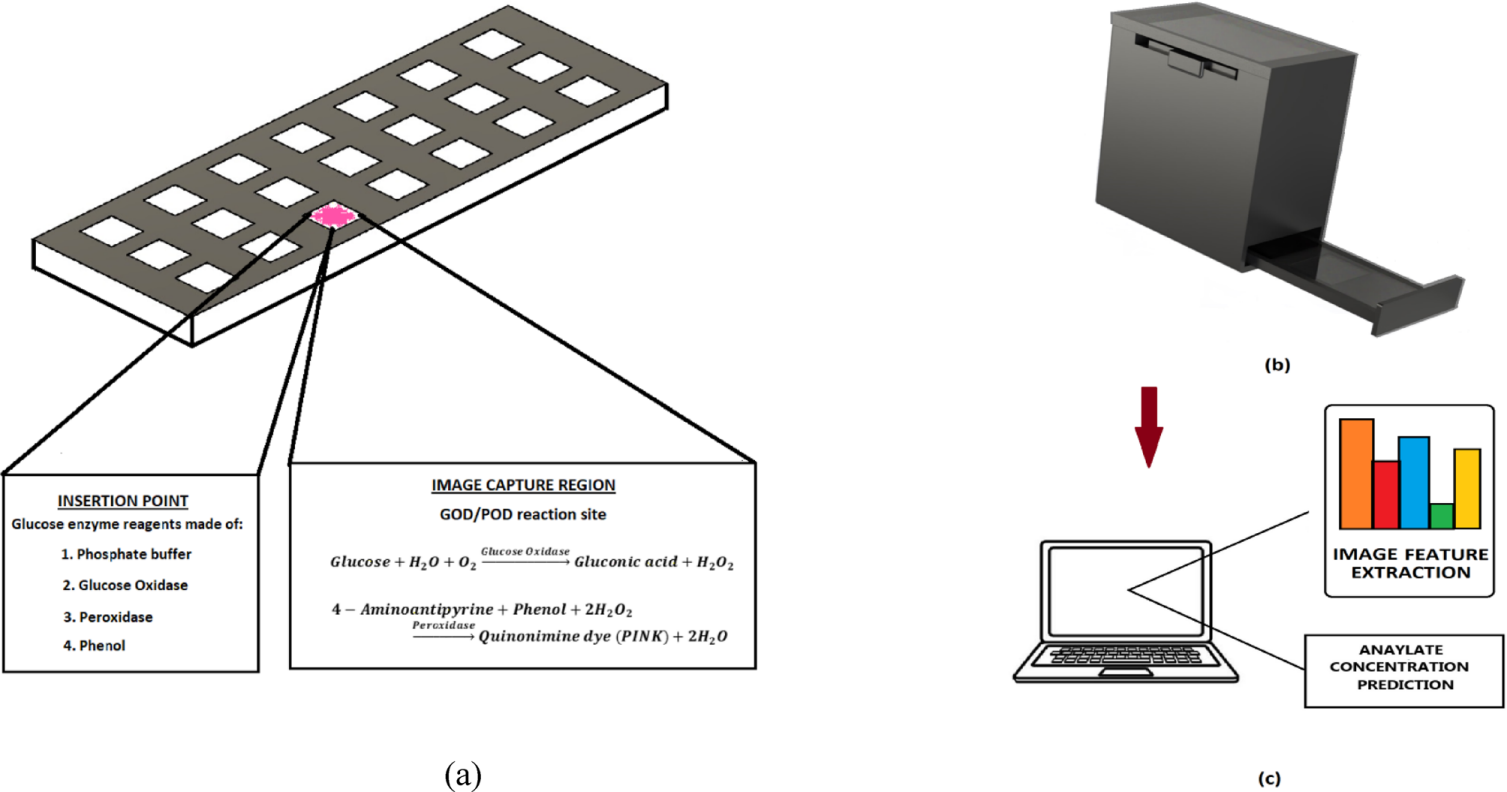
Overview of the present study. (**a**) The functionality of the microfluidic chip is evaluated using the Glucose Oxidase–Peroxidase (GOD–POD) reaction for colorimetric glucose estimation. (**b**) An image capture device embedded with fixed camera and light source with the region of interest (ROI) identified using a box algorithm. (**c**) The captured glucose sample images are processed after data analysis, feature extraction/selection and provided as input to machine learning classifier to determine the corresponding glucose concentration levels. (The 3D model is generated using Autodesk Fusion 360 Version 2.0 software; https://www.autodesk.com/education/edu-software/overview#FSN).

**Fig. 2:**
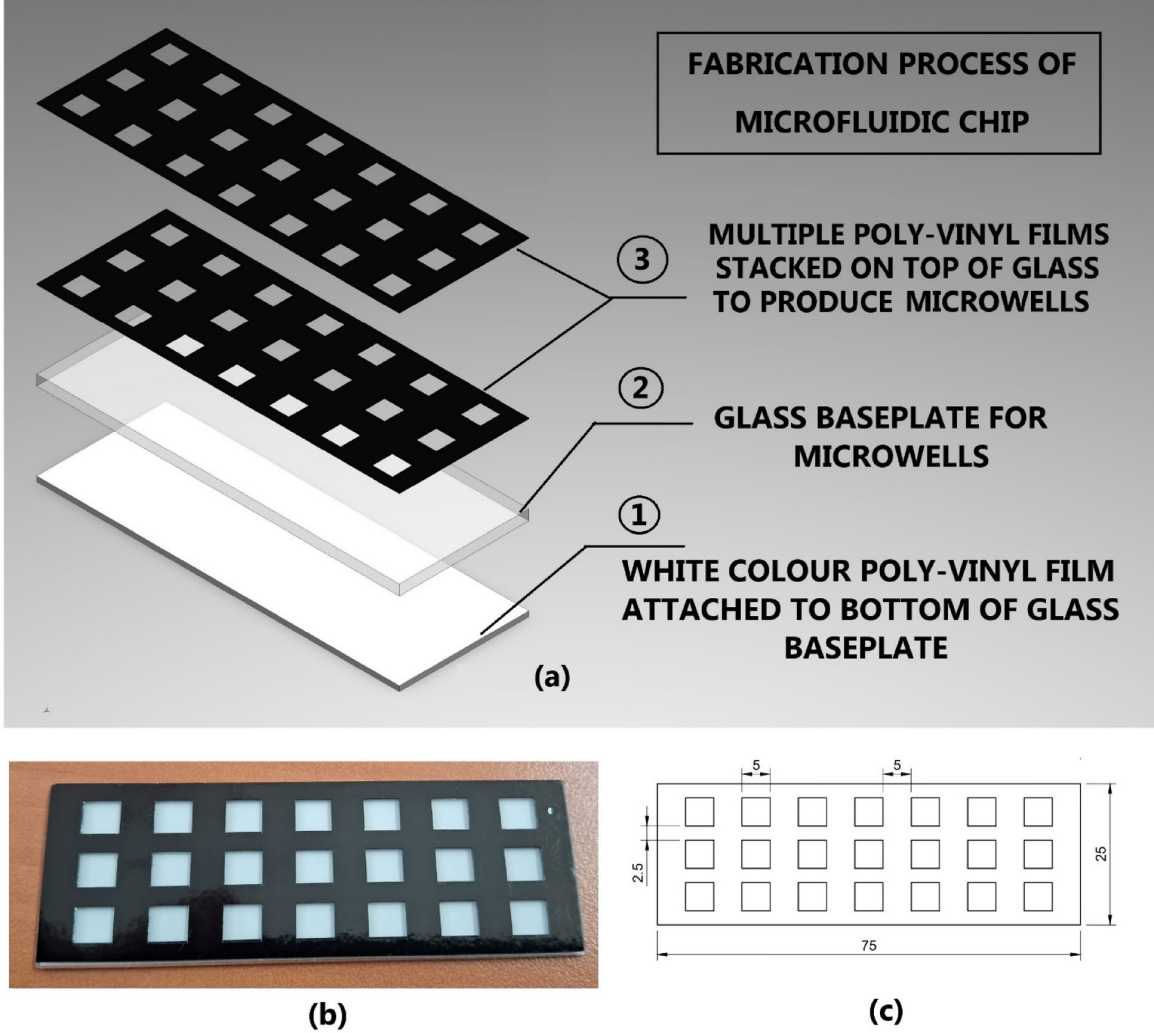
Illustration of the stepwise fabrication of a microfluidic chip utilizing an adhesive-coated polyvinyl film stack on a glass baseplate. (**a**) The device is composed of three distinct layers: (1) A white polyvinyl film layer adhered to the underside of the glass base, providing a high-contrast background for imaging; (2) A transparent glass baseplate onto which polyvinyl films are attached on the top surface; (3) One or more layers of adhesive-backed black vinyl films, cut precisely using a cutting plotter to create microwells, with the depth controlled by the number of layers. (**b**) An image of the fabricated microfluidic chip. (**c**) A two-dimensional schematic representation of the microfluidic chip with dimensional specifications in millimeters. (Image source: Kanchan et al.^38^).

An overview of the experimental framework is illustrated in Fig. 1. The microfluidic system employed in this investigation was fabricated through a multi-layered assembly of adhesive-backed polyvinyl chloride (PVC) films, as shown in Fig. 2. The structural design of the microfluidic channels was created using CorelDRAW Graphics Suite 2018 (Version 20.1.0.708), available at https://www.coreldraw.com, which was then used as the vector input for a precision cutting plotter. A rigid glass microscope slide (75 mm × 25 mm × 1 mm) functioned as the foundational substrate, ensuring stability and a uniform surface for microwell integration. The fabrication workflow involved strategic layering of adhesive-coated PVC films in white, black, and transparent variants. Initially, a white PVC film was dimensionally cut to conform to the glass slide and affixed to one of its surfaces, thereby establishing a high-contrast background conducive to image-based analysis. The next phase entailed precision cutting of black PVC film to delineate the internal architecture of the microfluidic device, comprising 21 microwells, each measuring 5 mm × 5 mm, as depicted in Fig. 2a. This structured black PVC film was subsequently adhered atop the glass substrate. To modulate the depth of the microfluidic channels, additional black PVC layers were stacked, culminating in a total depth of approximately 200 μm. Following complete assembly, rigorous quality control inspections were conducted to confirm the structural integrity and mitigate potential fluidic leakage at the interfacial junctions.

### Analytical methodology and reaction protocol

For glucose quantification, the Mybio Glucose Test Kit (Mylab Discovery Solutions Pvt. Ltd., India) was employed. This diagnostic kit contained a glucose-specific enzyme reagent, formulated with phosphate buffer, glucose oxidase (GOD), peroxidase (POD), 4-aminoantipyrine, and phenol, along with a reference glucose standard. The detection mechanism leveraged the enzymatic GOD-POD reaction pathway for glucose determination. To align with physiological glucose concentrations observed in normal glycemic individuals (fasting: 70–110 mg/dL; postprandial: up to 130 mg/dL), a series of calibration solutions were meticulously prepared using D-galactose (SRL Chemicals, India). The calibration curve was constructed by preparing standard glucose solutions with concentrations incrementing from 50 to 200 mg/dL in 10 mg/dL steps, resulting in a total of 16 reference samples.

To ensure precise volumetric dispensing and minimize systematic errors, all sample preparations were executed using calibrated micropipettes spanning volume ranges of 1.0–10 µL and 100–1000 µL. Spectrophotometric validation was conducted on the prepared glucose standards to verify concentration accuracy before application within the microfluidic platform. The microfluidic device was subsequently utilized to analyze glucose levels across the prepared samples. For point-of-care deployment, alternatives to conventional micropipettes could enhance usability and workflow efficiency. Potential approaches include the integration of pre-calibrated capillary tubes, single-use transfer pipettes, or droplet dispensers for consistent volumetric delivery. Additionally, embedding a microfluidic actuation system within the chip design could automate fluid handling, minimizing manual intervention while ensuring high precision in reagent and sample administration.

### Image capture device

**Fig. 3:**
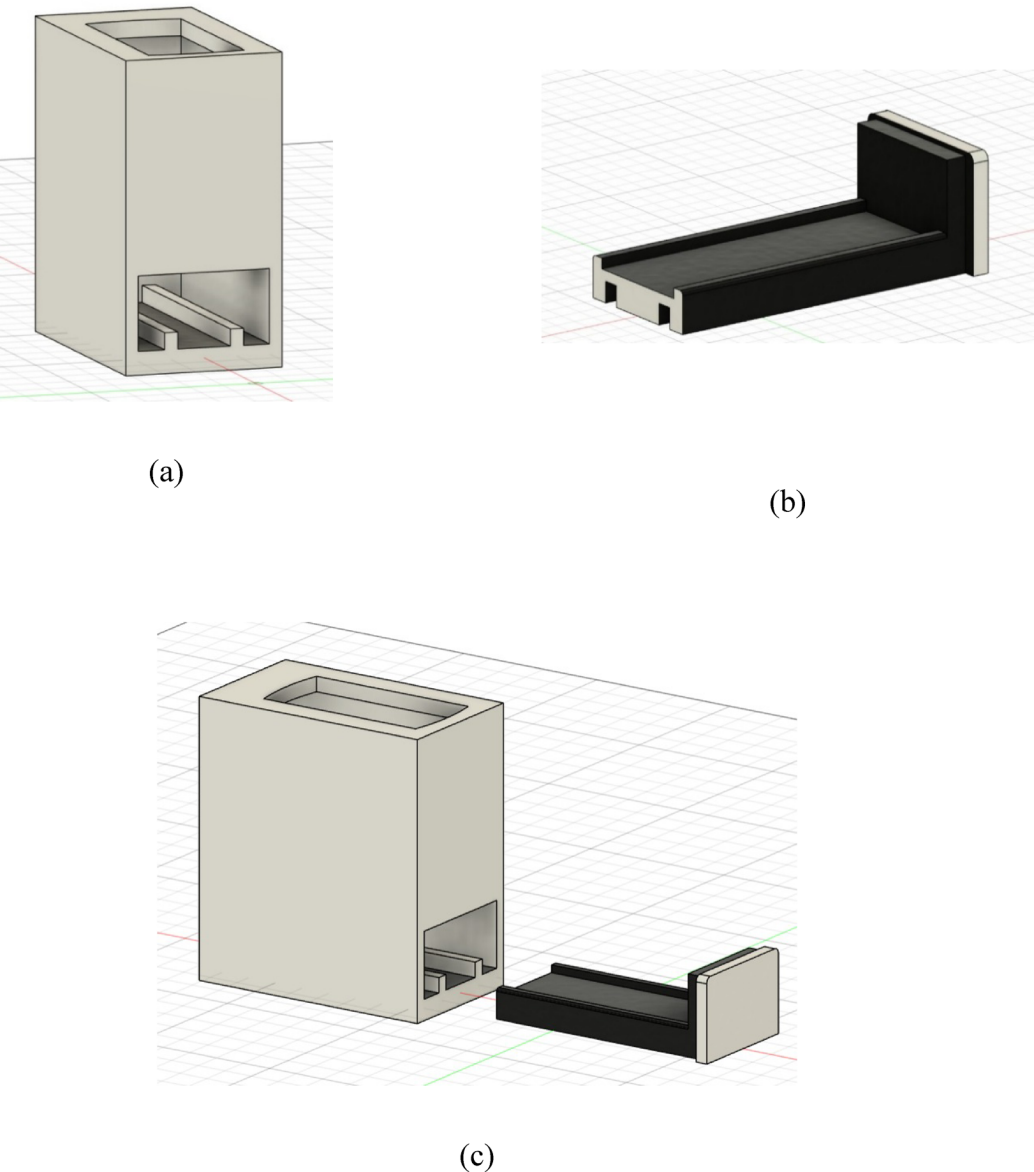
CAD model of the image capture device. (a) The main body consists of opening at top for fixing camera module and opening at lower portion for slider, (b) The microfluidic chip holder that slides inside the main body. (c) Combined view of main body and slider. (The 3D model is generated using Autodesk Fusion 360 Version 2.0 software; https://www.autodesk.com/education/edu-software/overview#FSN).

**Fig. 4:**
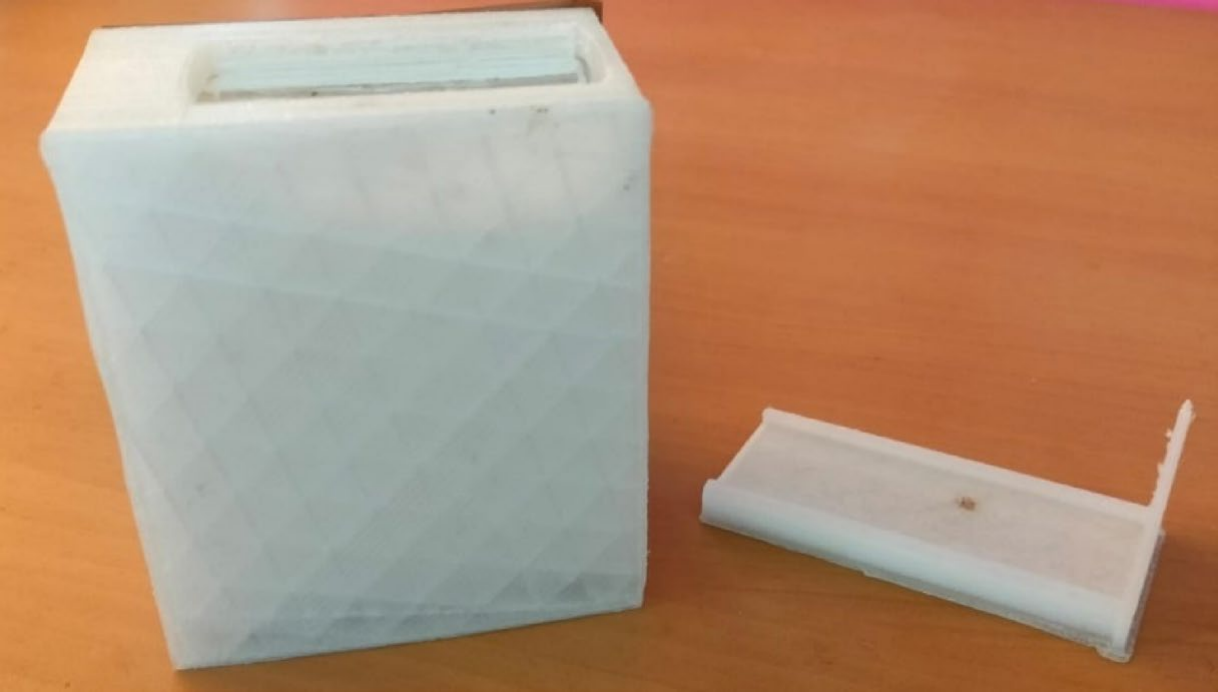
3D printed main body and slider with grooves for holding the microfluidic chip.

The CAD model of the image capture device used in the present study is shown in Fig. 3. The device is modelled using Fusion360 software. The 3D printer used for the fabrication process was Fracktal Volterra. The dimensions of the fabricated image capture device are 7.8 cm × 4.6 cm × 9 cm. The CAD model was sliced using Fracktory software and provided as input to the machine. In the fabrication process, Polycarbonate filament was used with 0.8 nozzle and 70% triangular pattern infill. The fabricated main body and microfluidic chip holding slider are shown in Fig. 4.

**Fig. 5:**
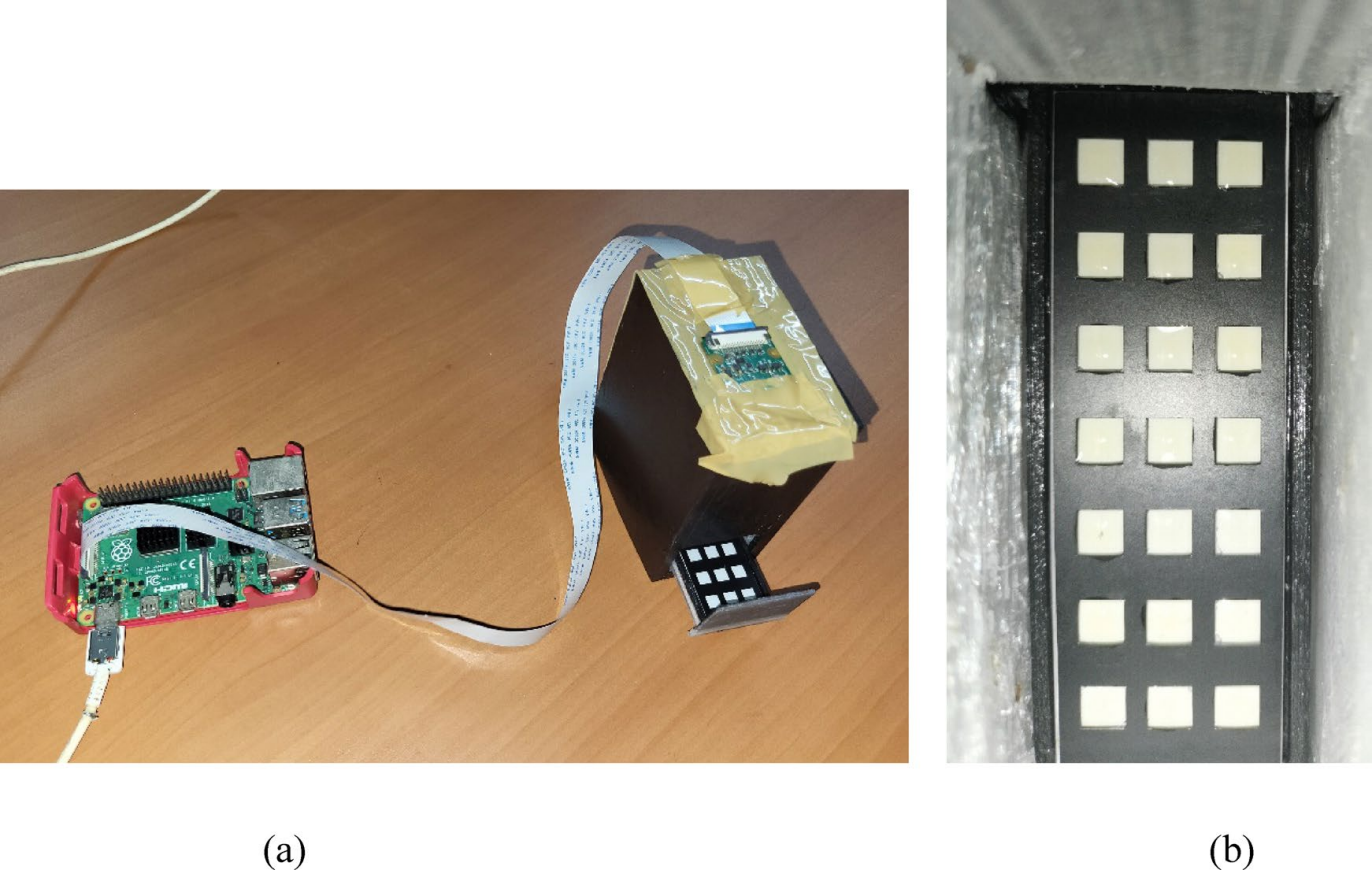
(**a**) The experimental setup consists of an image capture device embedded with a camera module and image processor. The microfluidic chip is positioned on the slider. (**b**) Sample image captured inside the device.

Figure 5 shows the experimental setup consisting of image capture device, microfluidic chip, camera and processor. A Raspberry Pi AI camera (Sony IMX500) sensor compatible with Raspberry Pi 4 processor is used in the present study. A 0.2 W Mini LED light powered by the same processor is fixed at the bottom of the device for better illumination and to maintain fixed light conditions. A single glucose concentration is prepared at a time and placed in 20 out of the 21 micro wells available in each microfluidic chip. A single main image is captured, and 20 images are developed from the main image by cropping the Region of Interest (ROI) using a box detection algorithm. Considering the fixed position of the camera and optimum lightning conditions, 80 images were captured for each concentration. This results in a dataset generation of 1280 images for glucose concentration detection.

### Data analysis and model evaluation

**Fig. 6:**
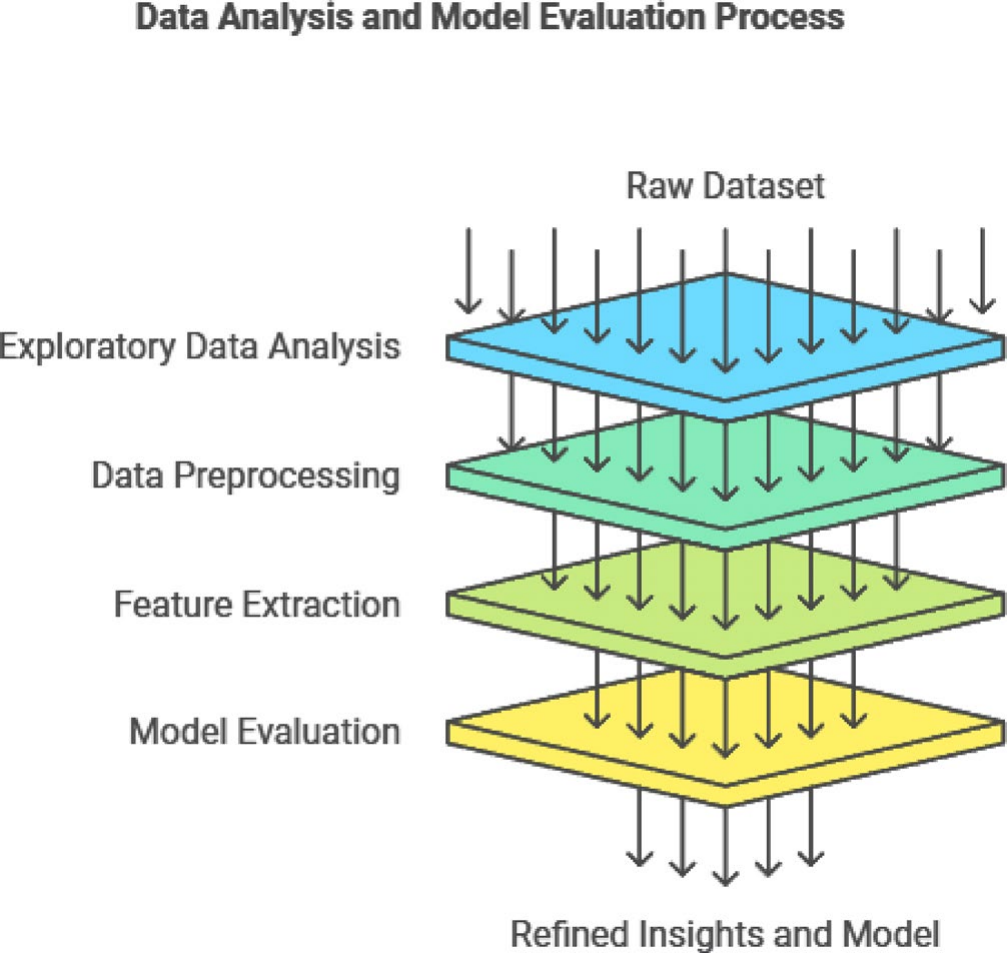
Schematic representation of the data analysis and model evaluation process, depicting key steps from raw data preprocessing to refined insights and model development.

The methodology adopted in this study consists of multiple steps, as shown in Fig. 6. First, an exploratory data analysis (EDA) was conducted, employing visualizations such as histogram, heatmap etc. The dataset distribution was examined to ensure a balanced representation of glucose concentration classes, preventing bias in model training. Pixel intensity histograms revealed variations in brightness and contrast, aiding in feature extraction and preprocessing optimization. Scaling was performed by normalizing pixel values to the range [0,1], ensuring uniform intensity representation across images. This step helps in stabilizing the learning process and improving convergence during training. Next, normalization was applied using the ImageNet, with mean ([0.485, 0.456, 0.406]) and standard deviation ([0.229, 0.224, 0.225]), standardizing pixel intensities and reducing variations due to lighting conditions. Additionally, all images were resized to 128 × 128 pixels, ensuring uniform input dimensions for the model while preserving critical features for glucose concentration estimation. Finally, the images were converted into PyTorch tensors, enabling efficient batch processing and GPU acceleration during training. These preprocessing steps collectively optimize feature extraction and improve model robustness for accurate glucose concentration classification.

### Feature extraction

Feature extraction plays a crucial role in capturing key characteristics of pixel intensity variations within images. A sliding window approach was employed to compute statistical features, ensuring that localized intensity variations were effectively analysed. The mean pixel intensity represents the average brightness within a region, serving as a fundamental descriptor of overall illumination differences across glucose concentration levels. Standard deviation and variance measure the dispersion and spread of intensity values, helping to identify contrast variations that may be linked to glucose-induced optical changes. Skewness evaluates the asymmetry of intensity distributions, indicating shifts in brightness dominance within glucose concentration samples, while kurtosis assesses the “tailedness” of intensity distributions, detecting outliers or unique intensity regions. Energy, defined as the sum of squared pixel values, provides an estimate of signal strength, aiding in detecting glucose-dependent intensity patterns. Entropy quantifies the randomness of pixel intensities, with higher entropy indicating greater complexity, potentially linked to variations in glucose levels. Finally, smoothness measures uniformity in intensity transitions, essential for distinguishing structured patterns from homogeneous regions in glucose-related images. These extracted features offer a comprehensive representation of intensity variations, texture properties, and statistical distributions, facilitating the development of an accurate classification model for glucose concentration estimation. Table 1 presents the significance of the extracted features used for glucose concentration classification.

**Table 1:** Feature extraction methods and their significance.

Feature	Significance
Mean (µ)	Represents the average brightness of an image region, distinguishing different glucose concentration levels based on illumination
Standard deviation (σ)	Measures contrast variations; higher values indicate greater intensity variation, useful for identifying texture differences
Variance (σ^2^)	Describes the spread of pixel intensities, aiding in texture differentiation linked to glucose-induced changes
Skewness (S)	Indicates asymmetry in intensity distribution, helping detect brightness dominance in glucose concentration samples
Kurtosis (K)	Measures “tailedness” of intensity distribution, identifying outliers or unique intensity regions associated with glucose levels
Energy (E)	Reflects overall intensity strength in an image, useful in detecting patterns influenced by glucose concentration
Entropy (H)	Captures randomness in intensity values; high entropy suggests complex textures, which may indicate glucose-induced optical variations
Smoothness (R)	Evaluates uniformity of pixel intensity transitions, helping differentiate structured and homogeneous regions

### Feature selection

Feature selection was performed to identify the most relevant features for glucose concentration classification, reducing dimensionality and improving model performance. To enhance model performance and interpretability, feature selection was carried out using wrapper methods, namely Forward Selection, Backward Elimination, and Recursive Feature Elimination (RFE). These techniques aim to identify the most relevant features that contribute significantly to glucose concentration classification while eliminating redundant or less informative ones. Forward Selection starts with no features and progressively adds the most impactful ones until model performance no longer improves. Backward Elimination begins with all features and iteratively removes the least significant ones based on their contribution to the model. Recursive Feature Elimination (RFE) systematically ranks features by training the model multiple times and eliminating the weakest contributors at each iteration. By applying these methods, the most discriminative features were selected, ensuring that the model achieves high accuracy while reducing computational complexity and overfitting. This optimized feature subset improves classification efficiency, leading to a more robust and interpretable glucose concentration prediction system.

**Fig. 7:**
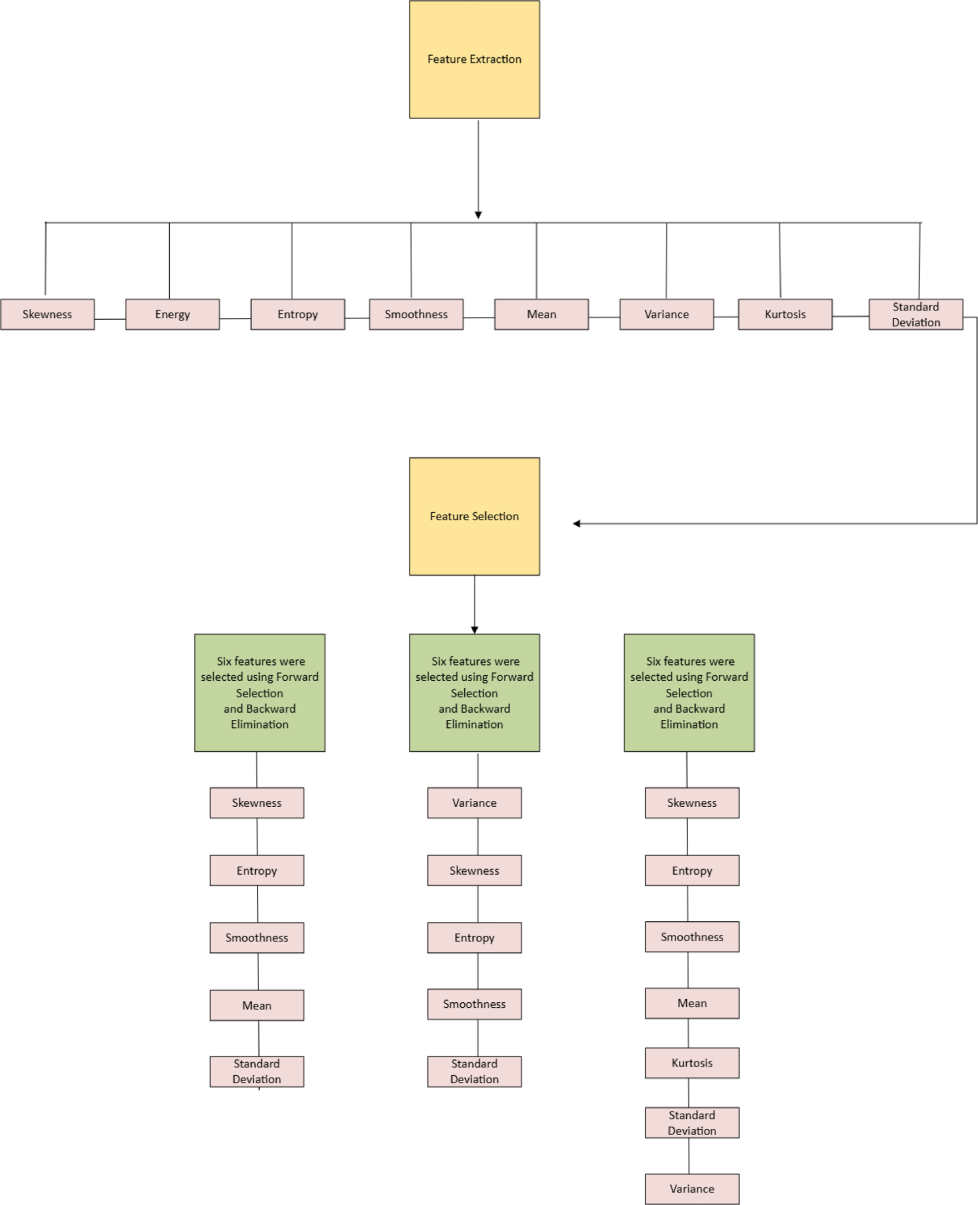
Overview of the feature extraction and selection process for glucose concentration classification.

Figure 7 illustrates the overall feature extraction and selection process for glucose concentration classification. Initially, a comprehensive set of features—skewness, energy, entropy, smoothness, mean, variance, kurtosis, and standard deviation—was extracted, capturing essential statistical and signal-based properties. To refine the feature set, three wrapper-based methods—Forward Selection, Backward Elimination, and Recursive Feature Elimination (RFE)—were applied. Forward Selection and Backward Elimination identified six key features across different trials, consistently selecting skewness, entropy, smoothness, mean, and standard deviation. Backward Elimination emphasized variance and kurtosis, while RFE ranked mean and smoothness as the most influential features for classification. This streamlined selection process enhances model interpretability and efficiency, improving glucose concentration prediction by retaining only the most informative attributes.

## Results and discussion

In our study, glucose detection was carried out using the glucose oxidase-peroxidase (GOD-POD) reaction. The colorimetric performance of the fabricated microfluidic chip was qualitatively validated prior to applying machine learning analysis. With 21 micro wells available, we were able to analyze all 16 glucose samples at the same time. During the reaction, glucose in the sample undergoes oxidation in the presence of glucose oxidase, generating gluconic acid and hydrogen peroxide. Peroxidase then catalyzes the oxidative coupling of 4-aminoantipyrine with phenol, resulting in the formation of a pink quinonimine complex. The intensity of this colored complex correlates directly with the glucose concentration in the sample. Visual inspection confirmed that the color intensity increased with glucose concentration, and color homogeneity across microwells was consistent for each sample. A representative image is shown in Fig. 5b. No leakage or gradient inconsistencies were observed, supporting the chip’s capability for uniform molecular sensing. These consistent, visually distinct outputs across concentrations provide a justified foundation for image-based machine learning classification.

### Exploratory data analysis (EDA)

**Fig. 8:**
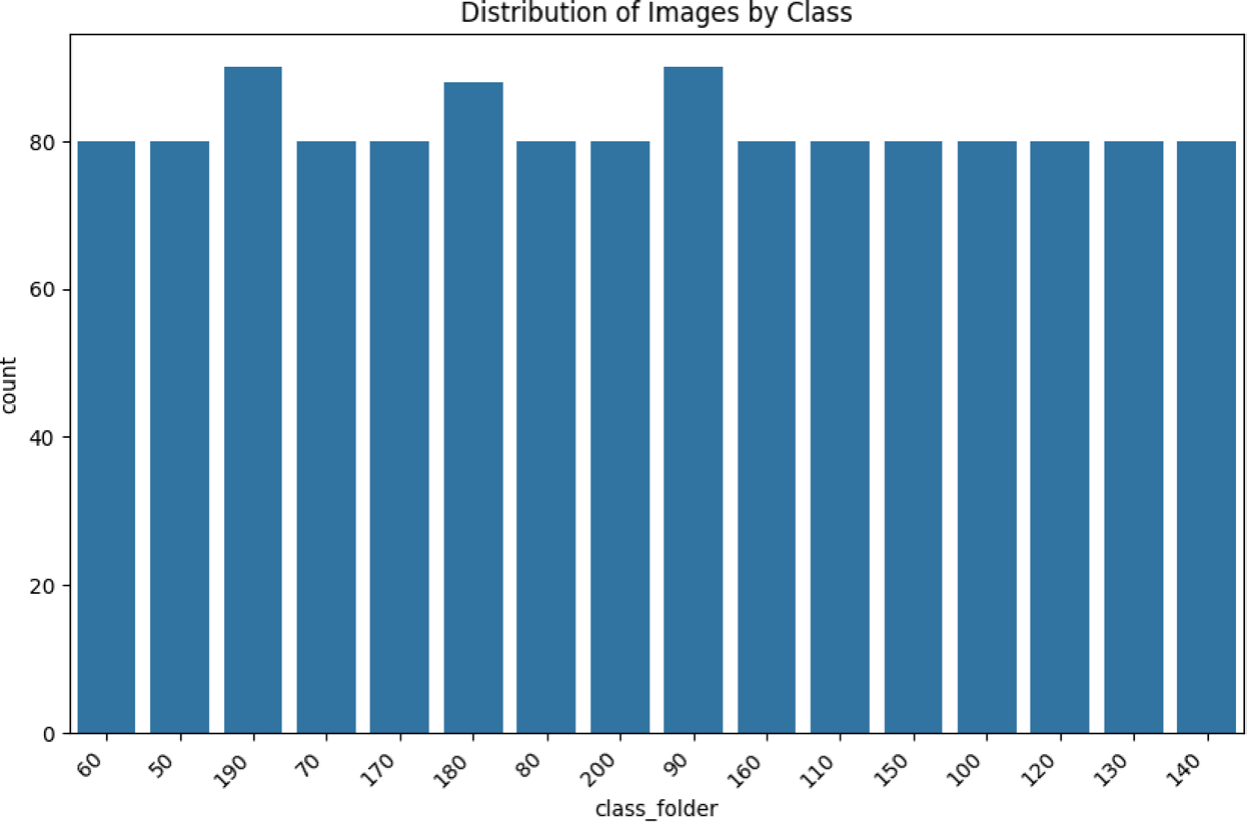
Distribution of images across different class labels.

As part of Exploratory Data Analysis (EDA), the dataset distribution was examined to ensure a balanced representation across different glucose concentration classes, as shown in Fig. 8. The histogram illustrates the number of images per class, confirming that the dataset does not exhibit significant class imbalance. This balance is crucial for training a machine learning model that generalizes well across different glucose concentration levels. A well-distributed dataset ensures that the model does not develop biases toward any particular class, thereby improving classification performance and robustness.

**Fig. 9:**
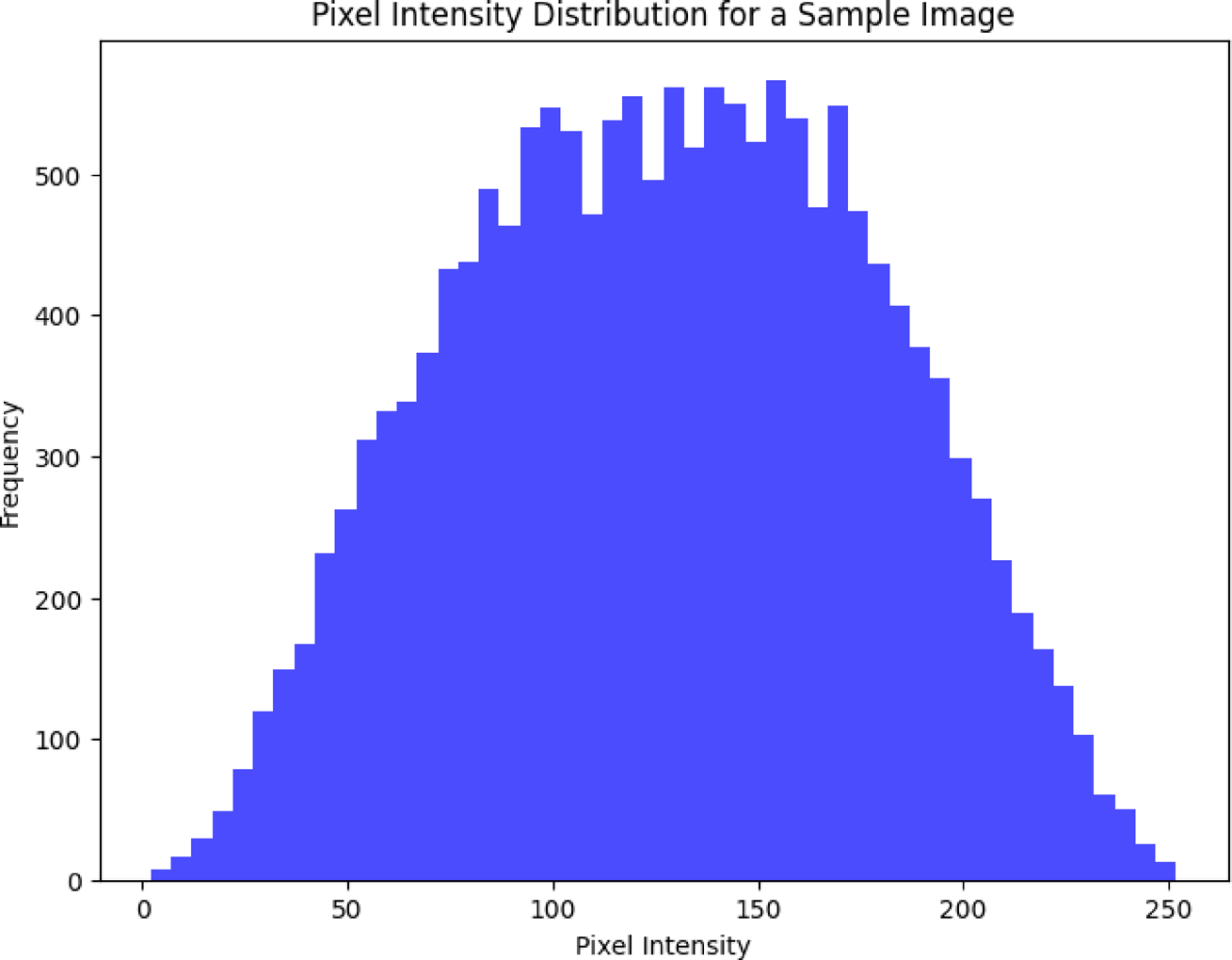
Pixel intensity distribution for a sample image, illustrating the variation in brightness levels across the image.

To further analyze the dataset characteristics, a pixel intensity distribution was plotted for a sample image, as shown in Fig. 9. This histogram provides insight into the brightness and contrast variations within an image, which are critical for feature extraction in glucose concentration estimation. A well-distributed pixel intensity range ensures that key features, such as brightness fluctuations and contrast differences, are adequately captured. This analysis aids in optimizing preprocessing techniques like contrast enhancement and normalization, improving feature quality, and ultimately enhancing the model’s ability to differentiate glucose levels.

**Fig. 10:**
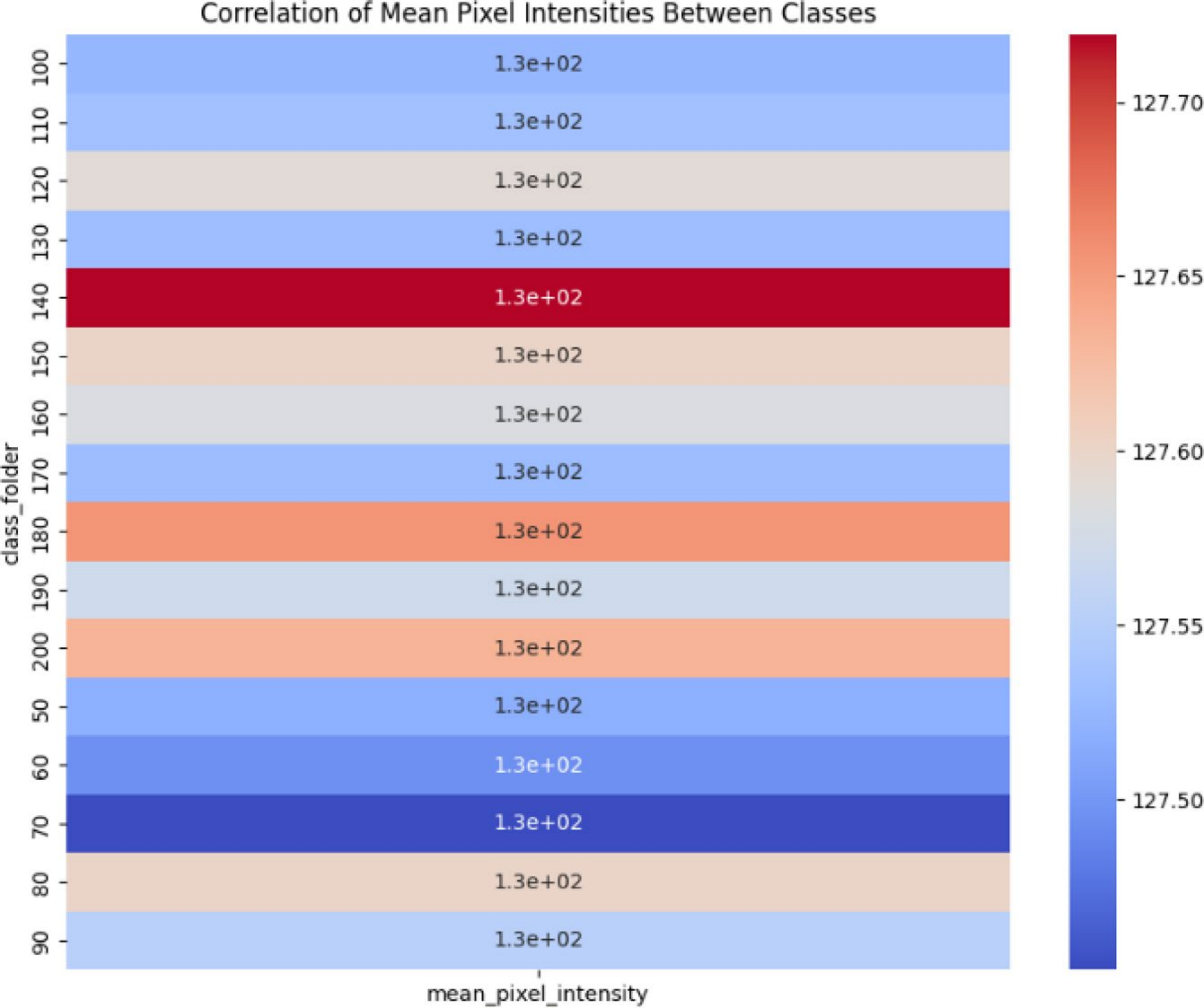
Correlation of Mean pixel intensities between classes.

To analyze the relationship between image brightness and glucose concentration levels, Fig. 10 presents a heatmap visualizing the mean pixel intensities across different glucose concentration classes. The varying color intensities suggest that differences in image brightness may correspond to glucose concentration variations, highlighting potential correlations. This visualization aids in understanding whether brightness serves as a distinguishing factor in classification, thereby influencing feature selection and preprocessing strategies for the machine learning model. The observed patterns reinforce the importance of pixel intensity-based features for glucose concentration estimation.

**Fig. 11:**
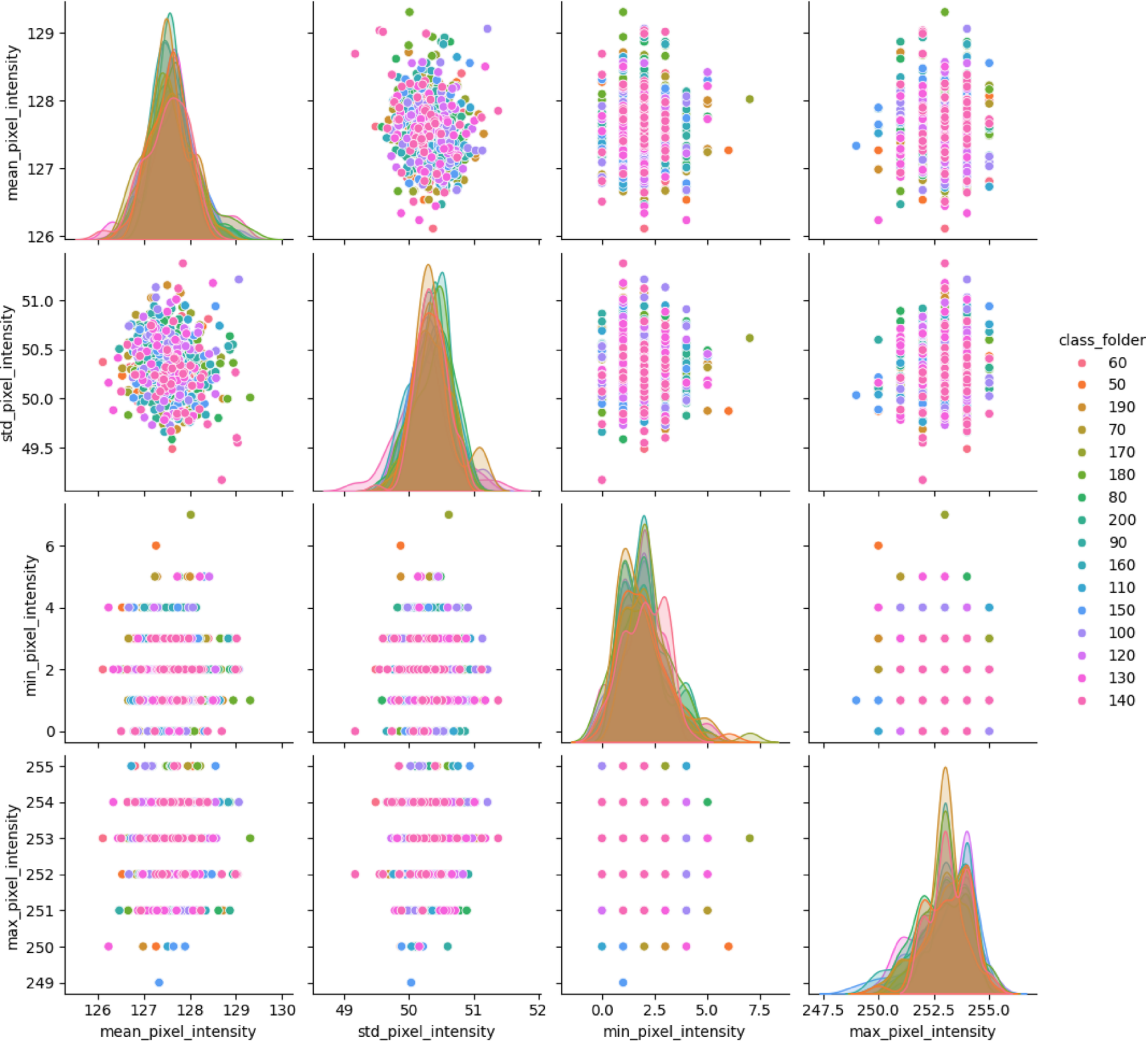
Pairwise relationships of pixel intensity features across classes.

Figure 11 presents a pairplot depicting the relationships between key pixel intensity features—mean, standard deviation, minimum, and maximum—across different glucose concentration classes. The diagonal plots show density distributions, indicating class-wise variations in pixel intensities, while the scatter plots reveal inter-feature correlations and potential clustering trends. This visualization helps assess class separability and identify the most informative intensity metrics for glucose concentration estimation, which is critical for enhancing model accuracy.

**Fig. 12:**
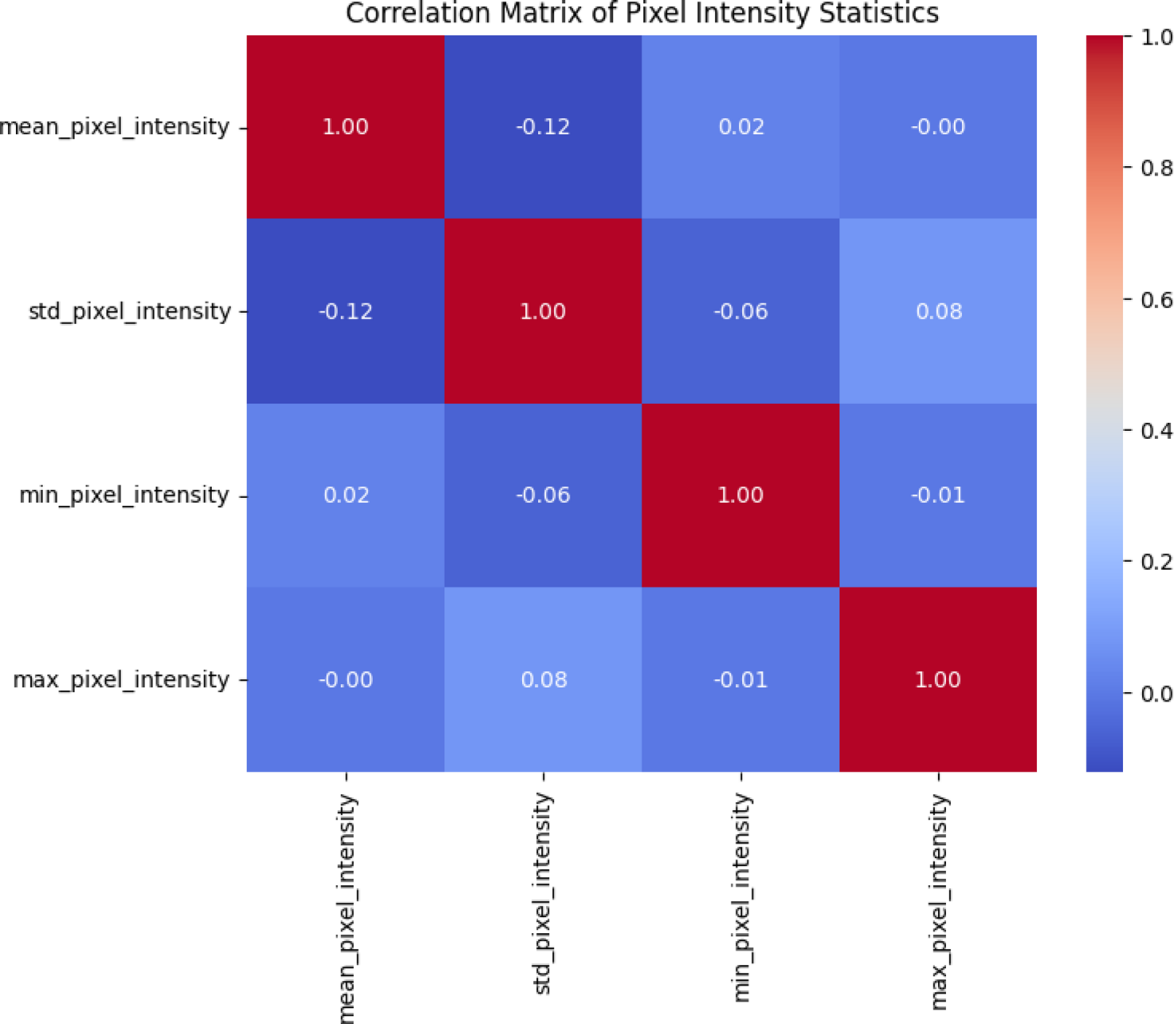
Correlation Matrix of Pixel Intensity Statistics.

Figure 12 presents a correlation matrix of pixel intensity statistics, illustrating the relationships between mean, standard deviation, minimum, and maximum intensity values. The heatmap visually represents the strength and direction of these correlations, with red indicating strong positive correlations and blue representing weak or negative associations. The low correlation values between these intensity features suggest that they provide largely independent information, minimizing redundancy and ensuring their utility as distinct features for glucose concentration estimation. This analysis validates the selection of diverse pixel intensity features for training an effective machine learning model.

### Feature extraction, selection and classification

Feature Extraction involved using statistical metrics like mean, standard deviation, variance, skewness, and entropy. These features help in distinguishing intensity variations and textures, aiding accurate glucose level classification. The distribution of various statistical features extracted from the dataset, which play a crucial role in glucose concentration classification can be found in Fig. A1 in Supporting Information.

Feature selection was performed to identify the most relevant features for glucose concentration classification, reducing dimensionality and improving model performance. To enhance model performance and interpretability, feature selection was carried out using wrapper methods, namely Forward Selection, Backward Elimination, and Recursive Feature Elimination (RFE). These techniques aim to identify the most relevant features that contribute significantly to glucose concentration classification while eliminating redundant or less informative ones. Forward Selection starts with no features and progressively adds the most impactful ones until model performance no longer improves. Backward Elimination begins with all features and iteratively removes the least significant ones based on their contribution to the model. Recursive Feature Elimination (RFE) systematically ranks features by training the model multiple times and eliminating the weakest contributors at each iteration. By applying these methods, the most discriminative features were selected, ensuring that the model achieves high accuracy while reducing computational complexity and overfitting. This optimized feature subset improves classification efficiency, leading to a more robust and interpretable glucose concentration prediction system.

#### Forward selection

From Fig. A2 (Supporting Information section), it is evident that mean, standard deviation (stddev), skewness, entropy, and smoothness were identified as the most discriminative features for glucose classification. Each of these features holds equal importance in the final model, indicating their strong influence on the predictive capability of the classifier. The selected features encompass both statistical properties (mean, stddev, skewness) and signal characteristics (entropy, smoothness), demonstrating a well-balanced representation of the data. The inclusion of these features enhances model robustness by improving classification accuracy while minimizing redundant or less informative attributes. By focusing on these selected features, the classification model achieves better interpretability, reduced overfitting, and improved generalization to unseen data.

The results of ten-fold cross-validation provide a comprehensive evaluation of different classifiers in predicting the target variable using the selected features. The models analyzed include Decision Tree (DT), Random Forest (RF), Support Vector Machine (SVM), Neural Networks (NN), and K-Nearest Neighbors (KNN) is shown in Table 2. The evaluation metrics considered are accuracy, precision, recall (sensitivity), and F1-score. The findings indicate that the Random Forest classifier outperforms other models with the highest accuracy (0.8747) and precision (0.8914), followed closely by the Decision Tree classifier. In contrast, Support Vector Machine (SVM) and Neural Networks (NN) exhibit significantly lower performance, with accuracy scores of 0.1179 and 0.1921, respectively. The KNN classifier shows moderate performance but remains suboptimal compared to RF and DT. The superior performance of Random Forest and Decision Tree suggests that tree-based models are better suited for this classification task, likely due to their ability to handle complex decision boundaries and high-dimensional feature spaces effectively.

**Table 2:** Model performance metrics for forward feature selection.

Model	Accuracy	Precision	Recall (sensitivity)	F1-Score
Decision Tree (DT)	0.8617	0.8751	0.8617	0.8607
Random Forest (RF)	**0.8747**	**0.8914**	**0.8747**	**0.8744**
Support Vector Machine (SVM)	0.1179	0.3190	0.1179	0.0981
Neural Network (NN)	0.1921	0.2087	0.1921	0.1801
K-Nearest Neighbours (KNN)	0.4952	0.5216	0.4952	0.4687

**Fig. 13:**
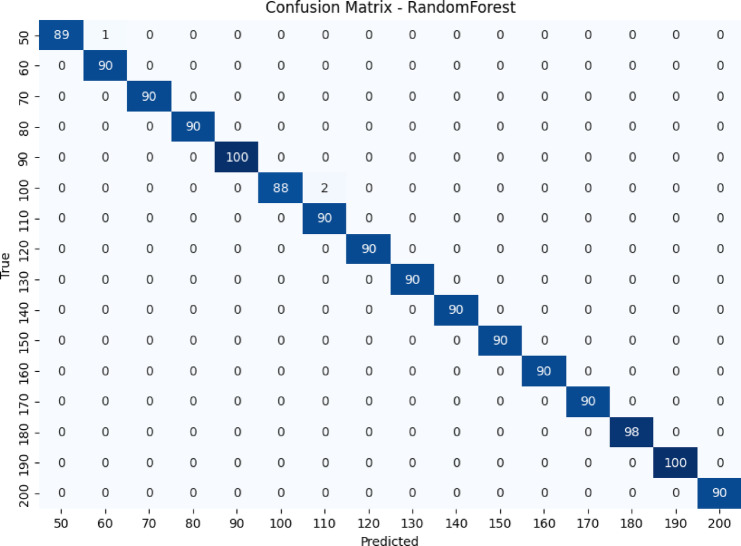
Confusion matrix of the random forest classifier.

The confusion matrix for the RF classifier provides a detailed assessment of the model’s classification performance as shown in Fig. 13. The matrix indicates a high level of accuracy, with most true labels correctly classified. Each class exhibits strong diagonal dominance, confirming that the model effectively differentiates between glucose concentration levels. Misclassifications are minimal, as evident from the limited presence of off-diagonal elements, which suggests that the RF model generalizes well to unseen data. A closer examination reveals that class 50 has a slight misclassification rate, where 1 instance was misclassified. Similarly, class 100 shows a minor misclassification with 2 incorrect predictions. However, the overall consistency of high values along the diagonal underscores the RF model’s robustness in handling feature variability. The strong predictive capability of RF, as seen in the confusion matrix, further supports its high cross-validation accuracy (0.8747) and precision (0.8914), making it the most reliable classifier among the evaluated models.

The results highlight the importance of selecting the right classification model for optimal performance. The low performance of SVM and NN may be attributed to the nature of the dataset or hyperparameter choices, while tree-based models like RF and DT effectively leverage feature importance to achieve higher accuracy and reliability.

The ROC curve in Fig. A3 (Supporting Information section) illustrates the classification performance of the Random Forest model, evaluated using ten-fold cross-validation. The curve represents the trade-off between the true positive rate (sensitivity) and the false positive rate, providing insights into the model’s discriminative ability. The model achieves a high mean Area Under the Curve (AUC) of 0.9509, indicating strong classification performance with minimal false positives. The blue curve demonstrates the stability of the model across different folds, consistently maintaining a high true positive rate. The dashed diagonal line represents random guessing, and the significant deviation of the ROC curve from this line further validates the effectiveness of the Random Forest classifier in glucose concentration classification.

#### Backward selection

Figure A4 (Supporting Information section) illustrates the features retained after the backward selection process. The selected features include variance, skewness, kurtosis, entropy, and smoothness, which were identified as the most relevant predictors for glucose concentration classification. The retention of variance and skewness suggests that fluctuations in signal characteristics play a crucial role in distinguishing different glucose levels. Kurtosis, which captures the tailedness of the distribution, provides additional insight into signal variations. Entropy, a measure of signal complexity, and smoothness, which represents signal stability, further contribute to the classification performance by capturing essential patterns in the data. The backward selection approach ensures that only the most influential features are retained, reducing model complexity while maintaining optimal performance. This step enhances the interpretability of the classification model and minimizes the risk of overfitting, thereby improving its generalization to unseen data.

Among the classifiers, Random Forest (RF) achieved the highest cross-validation accuracy of 87.13%, followed closely by the Decision Tree (DT) with 86.72% accuracy as shown in Table 3. Both models exhibited high precision and recall values, confirming their robustness in classification. Support Vector Machine (SVM) and Neural Networks (NN) underperformed significantly, with accuracies of 12.40% and 18.73%, respectively, indicating that these models struggled with feature discrimination after backward selection. K-Nearest Neighbors (KNN) demonstrated moderate performance, with an accuracy of 50%, higher than SVM and NN but lower than DT and RF.

**Table 3:** Model performance with backward feature selection.

Model	Accuracy	Precision	Recall (sensitivity)	F1-Score
Decision Tree (DT)	0.8672	0.8835	0.8672	0.8665
Random Forest (RF)	0.8713	0.8884	0.8713	0.8710
Support Vector Machine (SVM)	0.1240	0.3101	0.1240	0.1047
Neural Network (NN)	0.1873	0.1967	0.1873	0.1768
K-Nearest Neighbours (KNN)	0.5000	0.5109	0.5000	0.4642

The results indicate that tree-based models, particularly RF and DT, are the most suitable classifiers for glucose concentration classification, as they consistently achieved high accuracy and balanced performance across precision, recall, and F1-score. The poor performance of SVM and NN suggests that the selected feature subset might not be well-suited for these models, possibly due to their sensitivity to feature scaling and high-dimensional space representations.

**Fig. 14:**
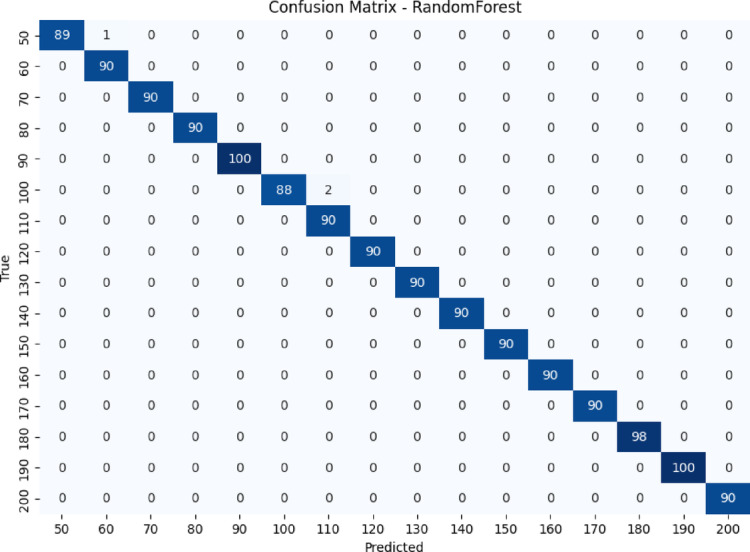
Confusion matrix for the Random Forest (RF) model.

The confusion matrix for the RF model, as shown in Fig. 14, highlights its strong classification performance, with a clear diagonal dominance indicating high accuracy across glucose concentration levels. Most classes exhibit near-perfect classification, such as 90/90 or 100/100, confirming the model’s robustness. Minor misclassifications are observed, including one instance in the 89 correctly classified, 1 misclassified and two in the 88 correctly classified, 2 misclassified, suggesting slight overlaps in feature space for adjacent glucose levels. Despite these small errors, the overall low misclassification rate reinforces RF as the most effective classifier, with high precision and recall across all categories. This analysis aligns with prior cross-validation results, further validating RF’s reliability for glucose concentration estimation, with potential improvements possible through hyperparameter tuning and additional training data.

The Receiver Operating Characteristic (ROC) curve for the Random Forest (RF) model demonstrates its ability to distinguish between different glucose concentration levels. The curve, generated using 10-fold cross-validation (Fig. A5 (Supporting Information section)), shows consistently high performance across multiple folds. The Mean AUC (Area Under the Curve) of 0.9450 indicates a strong discriminatory capability, with the RF model significantly outperforming random guessing (dashed line). The steep rise in the initial segment of the curve suggests that the model achieves high sensitivity while maintaining a low false positive rate, further confirming its robustness in classification tasks.

#### Recursive feature elimination (RFE)

Figure A6 (Supporting Information section) presents the feature rankings obtained through RFE for glucose concentration classification. The ranking indicates the relative importance of each feature, with mean and smoothness emerging as the most influential. Other features such as standard deviation (stddev), variance, skewness, kurtosis, energy, and entropy also contribute to the classification but with varying degrees of significance. The identification of these key features aids in optimizing the model while reducing computational complexity.

To evaluate the impact of recursive feature selection (RFS) on model performance, multiple machine learning algorithms were tested using cross-validation. The results for each model are summarized in Table 4. The Random Forest (RF) model demonstrated the highest classification performance, achieving a cross-validation accuracy of 0.8699, along with a precision of 0.8846 and an F1-score of 0.8692. The Decision Tree (DT) model followed closely, with a cross-validation accuracy of 0.8617 and an F1-score of 0.8617. In contrast, Support Vector Machine (SVM) and Neural Network (NN) performed poorly, with SVM yielding a significantly low cross-validation accuracy of 0.1308 and an F1-score of 0.1148, indicating ineffective classification capability in this scenario. The K-Nearest Neighbours (KNN) model achieved a moderate cross-validation accuracy of 0.5075, with a precision of 0.5337 and an F1-score of 0.4808. These results indicate that tree-based models, particularly Random Forest, remain the most reliable classifiers for the dataset, reinforcing their suitability for classification.

**Table 4:** Model performance with recursive feature selection.

Model	Accuracy	Precision	Recall (sensitivity)	F1-Score
Decision Tree (DT)	0.8617	0.8797	0.8617	0.8617
Random Forest (RF)	0.8699	0.8846	0.8699	0.8692
Support Vector Machine (SVM)	0.1308	0.3229	0.1308	0.1148
Neural Network (NN)	0.2425	0.2605	0.2425	0.2380
K-Nearest Neighbors (KNN)	0.5075	0.5337	0.5075	0.4808

**Fig. 15:**
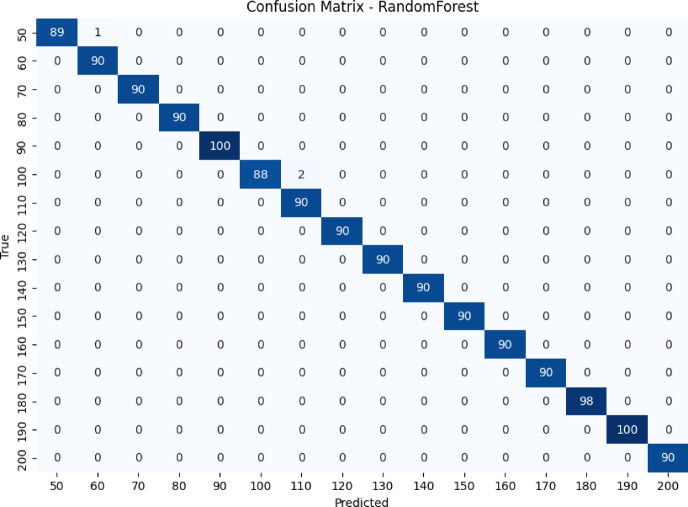
Confusion matrix for the Random Forest classifier in glucose concentration detection.

The confusion matrix for the Random Forest classifier, as shown in Fig. 15, demonstrates the model’s performance in classifying glucose concentration levels. The matrix reveals a strong diagonal dominance, indicating high classification accuracy, with most true labels correctly predicted. Misclassifications are minimal, with only a few instances of incorrect predictions appearing as off-diagonal elements. The model effectively distinguishes between different glucose concentration levels, with consistently high true positive rates across classes. These results further validate the robustness of the Random Forest model in handling glucose concentration detection, making it a promising approach for real-time monitoring and biomedical applications.

The Receiver Operating Characteristic (ROC) curve for the Random Forest classifier, shown in Fig. A7 (Supporting Information section), demonstrates its ability to distinguish between different glucose concentration levels. The curve is plotted for a ten-fold cross-validation setting, ensuring robustness in performance evaluation. The classifier achieves a high mean Area Under the Curve (AUC) value of 0.9509, indicating strong discrimination ability with minimal false positives. The consistent performance across folds, as evident from the tightly clustered ROC curves, further validates the model’s reliability in glucose concentration detection.

## Conclusion

The findings of this study highlight the successful development of a cost-effective microfluidic analytical chip, designed using a glass baseplate and layered polyvinyl films cut with precision using a cutting plotter. Polyvinyl adhesive films were selected due to their low cost, commercial availability and ease of patterning using a desktop cutting plotter. Their inherent adhesive nature enabled precise, leak-free bonding between layers. The glass baseplate was chosen for its high optical transparency, inertness to chemical reactions, and structural rigidity, which together ensured uniform light transmission and image clarity. This combination supports reliable colorimetric sensing and is suitable for scalable, low-cost Point-of-Care implementation. This innovative fabrication method enables rapid prototyping while optimizing the microfluidic environment for enzyme-reagent interactions essential for colorimetric detection. The chip is integrated into a custom-designed 3D-printed imaging device, equipped with a fixed-focus camera and controlled lighting, ensuring consistent image capture.

A machine learning-driven analytical framework was established for glucose concentration estimation, leveraging image-based feature extraction. Key numerical and pattern-based features, including mean pixel intensity, standard deviation, skewness, and entropy, were identified as crucial contributors to model performance. Through training on an extensive dataset, the models achieved efficient colorimetric detection without reliance on predefined calibration curves. Among the evaluated classifiers—Decision Tree (DT), Random Forest (RF), Support Vector Machine (SVM), Neural Networks (NN), and K-Nearest Neighbors (KNN)—tree-based models exhibited superior performance, with Random Forest emerging as the most effective. The RF model demonstrated the highest classification accuracy (87.47%) and precision (89.14%), supported by a robust confusion matrix and ROC curve analysis, yielding an AUC of 0.9509.

These results emphasize the viability of image-based glucose monitoring as a scalable, cost-effective, and non-invasive approach for real-time biomedical analysis. The demonstrated machine learning framework, coupled with the microfluidic imaging system, presents a promising alternative to conventional methods, paving the way for accessible and efficient diagnostic solutions.

## Supplementary Information

Below is the link to the electronic supplementary material.

Supplementary Material 1

## Data Availability

Data sets generated during the current study are available from the corresponding author upon reasonable request.
