# Supplementary material for: Development of colorimetric and machine learning based accurate glucose detection platform for point of care applications
Source: Sci Rep. 2026 May 20;16:22885. doi: 10.1038/s41598-026-54054-6 (PMC13389055; doi:10.1038/s41598-026-54054-6)
Supplement: Supplementary file 1 — Supplementary Material 1 [file 41598_2026_54054_MOESM1_ESM.pdf]

## SUPPORTING INFORMATION

The distribution of various statistical features extracted from the dataset, which play a crucial role in glucose concentration classification can be found in Figure A1.

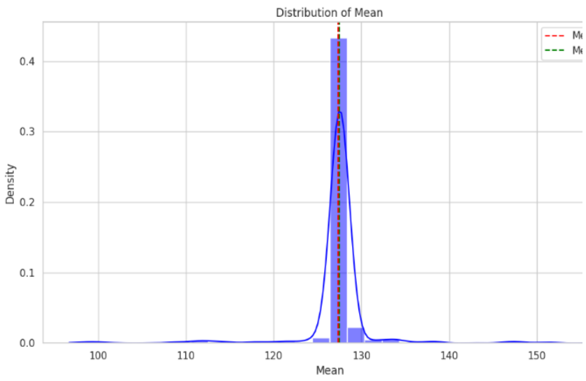


(a)

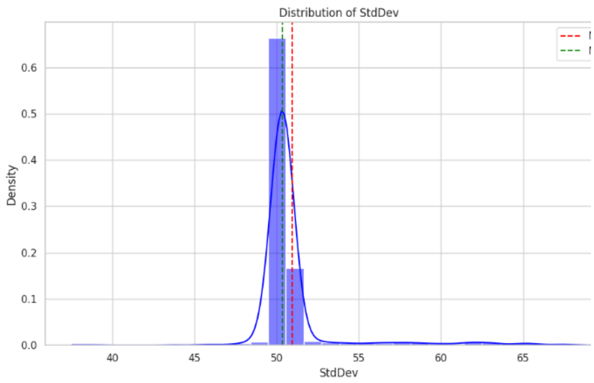


(b)

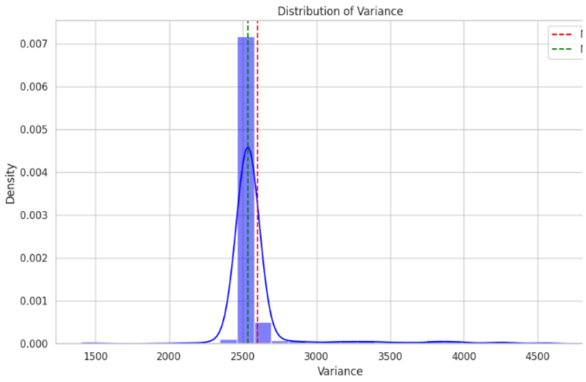


(c)

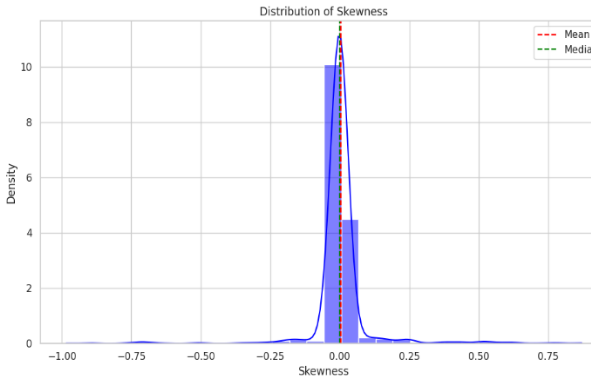


(d)

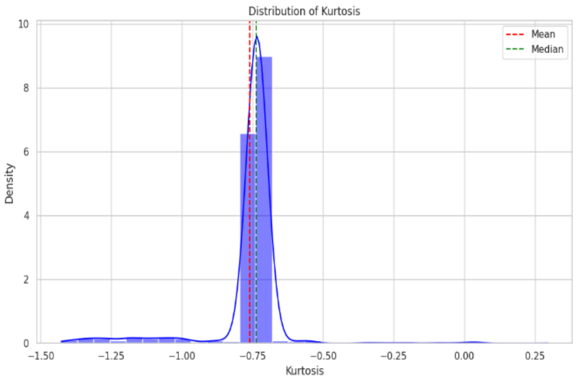


(e)

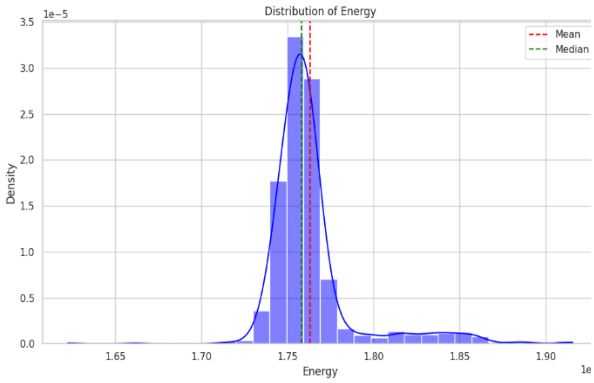


(f)

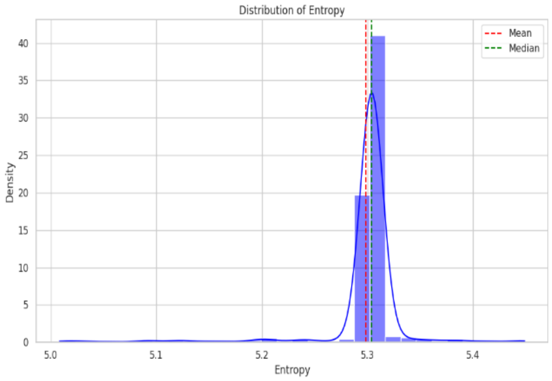


(g)

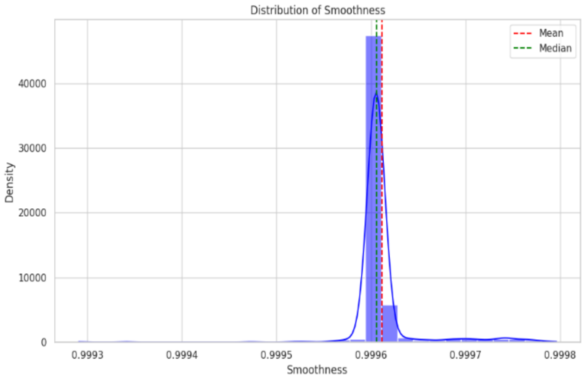


(h)

Fig. A1: Distribution of statistical features extracted from the images. (a) Mean intensity distribution, (b) Standard deviation of intensity values, (c) Variance, (d) Skewness, (e) Kurtosis, (f) Energy, (g) Entropy and (h) Smoothness. Dashed lines represent the mean (red) and median (green) values, providing insights into the statistical properties of the dataset.

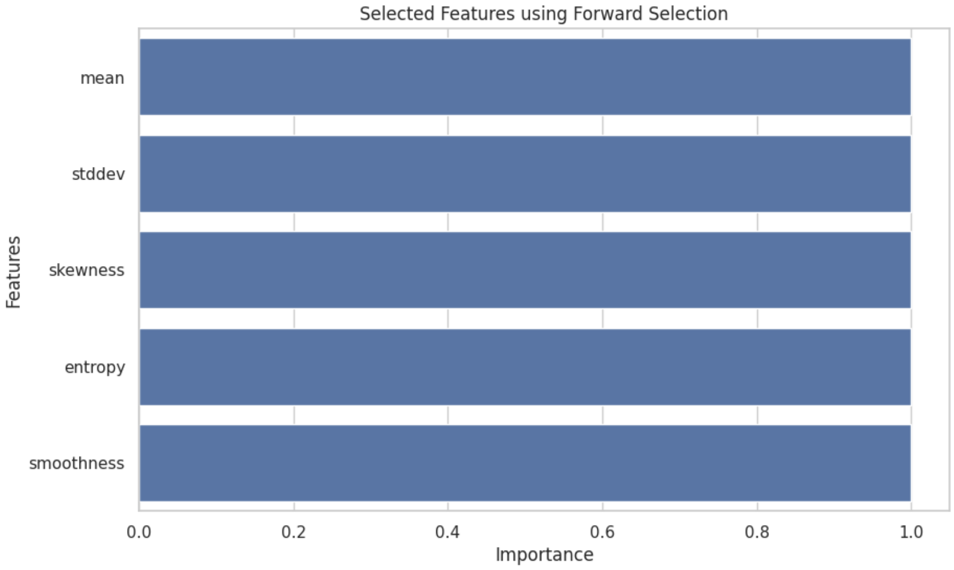


Fig. A2: Selected Features Using Forward Selection

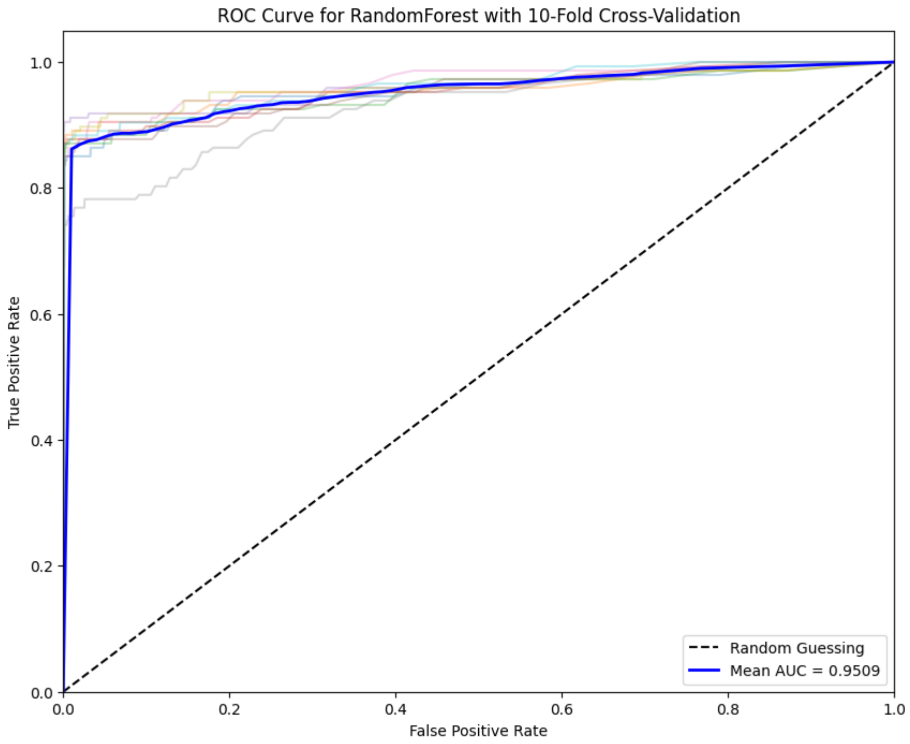


Fig. A3: ROC curve for the Random Forest model with 10-fold cross-validation

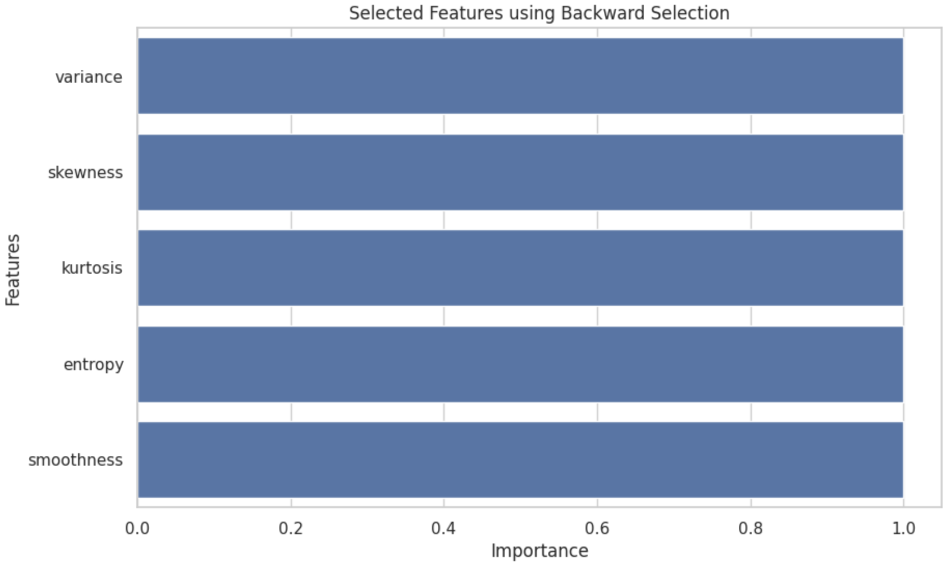


Fig. A4: Selected features using backward selection

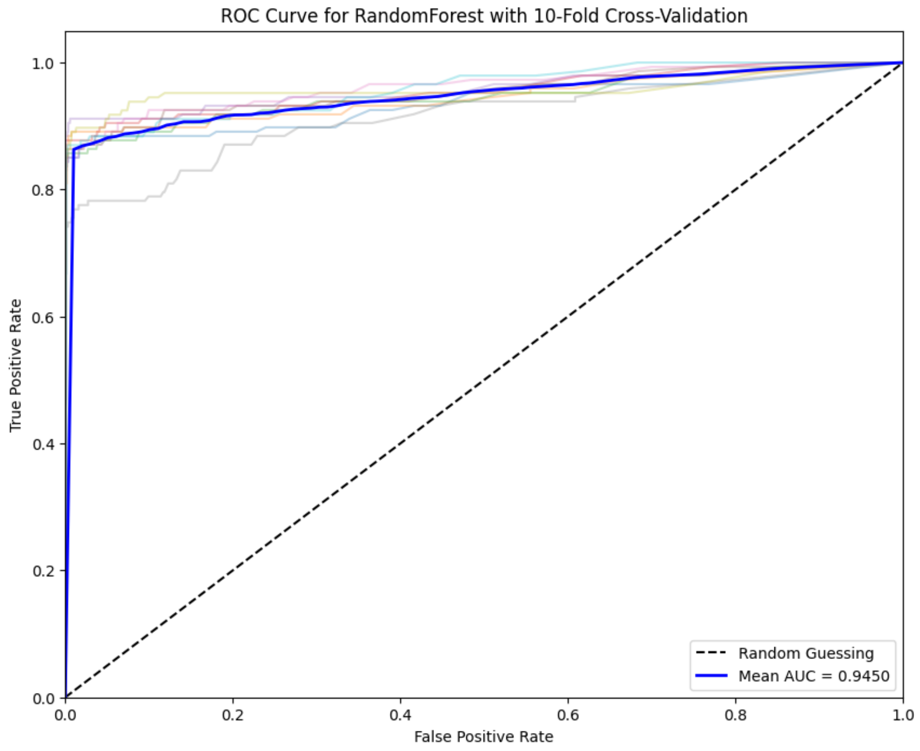


Fig. A5: ROC curve for the Random Forest model with 10-fold cross-validation with BFS.

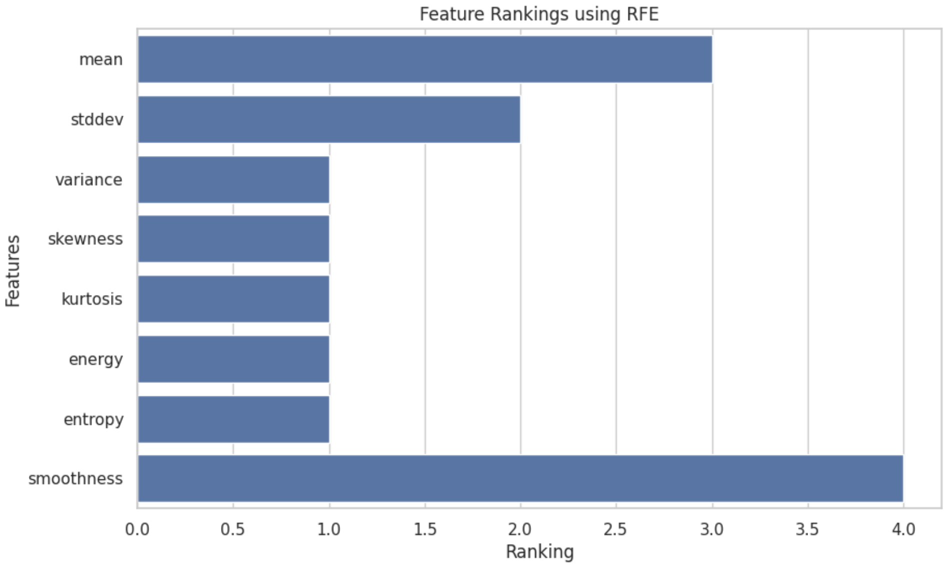


Fig. A6: Feature rankings using Recursive Feature Elimination (RFE)

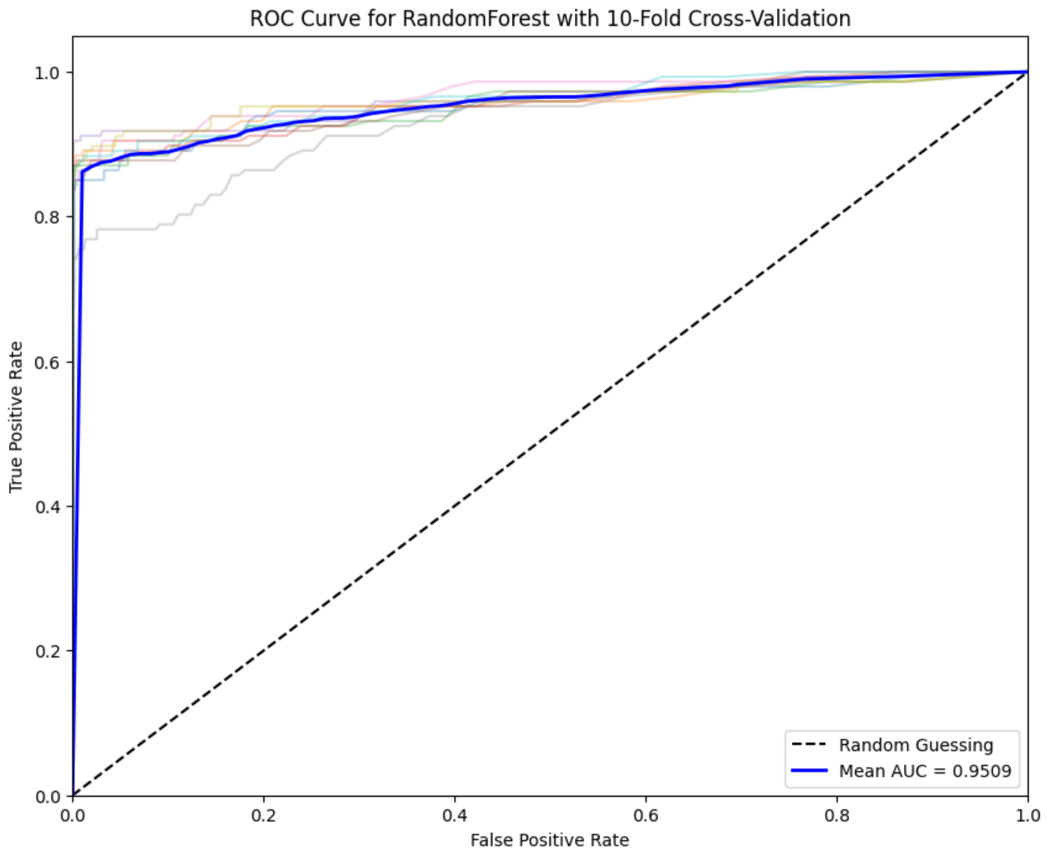


Fig. A7: ROC curve for the Random Forest model with 10-fold cross-validation with RFE.
